# EphB3 receptors function as dependence receptors to mediate oligodendrocyte cell death following contusive spinal cord injury

**DOI:** 10.1038/cddis.2015.262

**Published:** 2015-10-15

**Authors:** Y Tsenkina, J Ricard, E Runko, M M Quiala- Acosta, J Mier, D J Liebl

**Affiliations:** 1 The Miami Project to Cure Paralysis, The Department of Neurosurgery, Miller School of Medicine, The University of Miami, Miami, FL, USA; 2 Department of Biology, Drexel University, Philadelphia, PA, USA

## Abstract

We demonstrate that EphB3 receptors mediate oligodendrocyte (OL) cell death in the injured spinal cord through dependence receptor mechanism. OLs in the adult spinal cord express EphB3 as well as other members of the Eph receptor family. Spinal cord injury (SCI) is associated with tissue damage, cellular loss and disturbances in EphB3-ephrinB3 protein balance acutely (days) after the initial impact creating an environment for a dependence receptor-mediated cell death to occur. Genetic ablation of EphB3 promotes OL survival associated with increased expression of myelin basic protein and improved locomotor function in mice after SCI. Moreover, administration of its ephrinB3 ligand to the spinal cord after injury also promotes OL survival. Our *in vivo* findings are supported by *in vitro* studies showing that ephrinB3 administration promotes the survival of both oligodendroglial progenitor cells and mature OLs cultured under pro-apoptotic conditions. In conclusion, the present study demonstrates a novel dependence receptor role of EphB3 in OL cell death after SCI, and supports further development of ephrinB3-based therapies to promote recovery.

Spinal cord injury (SCI) leads to a severe functional disability affecting approximately 10 000 new people every year in the United States.^1^ The associated functional impairment in SCI patients is largely due to regional cell death and interruption of cerebrospinal axonal pathways. The initial mechanical tissue damage is accompanied by an acute necrotic and apoptotic cell loss followed by progressive degenerative events that include apoptosis (i.e., programmed cell death), demyelination, axonal injury, inflammation, edema, hemorrhage, excitotoxicity and oxidative stress.^2, 3, 4, 5, 6^ In mammals, SCI-induced apoptosis can last for days to weeks after the initial insult, where myelin-producing oligodendrocytes (OLs) are highly susceptible. OL loss underlies progressive demyelination and axonal degeneration that severely impact sensorimotor functions following SCI.^2, 7, 8, 9, 10, 11, 12^ In human patients, OL apoptosis is observed as early as 3 h after SCI and persists for weeks,^13^ providing a strong rationale for examining the mechanisms leading to OL death in the injured spinal cord.

OL apoptosis can result from a number of injury-induced environmental influences where cell survival depends on the balance between pro-survival and pro-apoptotic signals.^4, 12, 14, 15^ In central nervous system (CNS) injury, membrane-bound death receptors are key players in tipping the balance toward cell death through activation of executioner caspases. The classic death receptor family is part of the tumor necrosis factor (TNF) receptor gene superfamily comprises tumor necrosis factor receptor 1 (TNFR1), CD95 (also called FasR), DR3, DR4, DR5 and DR6.^12, 16, 17, 18, 19, 20^ We have recently identified new pro-apoptotic receptors in the adult CNS, belonging to the dependence receptors family, that also activate terminal caspases but in the absence of their cognate ligand(s).^21, 22^

Dependence receptors were initially identified in cancer biology as important modulators of cancer progression.^23, 24, 25^ These receptors fulfill dual functions depending on their ligand availability. During normal development and tissue homeostasis, they interact with their respective ligand(s) to promote positive signals such as cell survival, migration and differentiation. However, when deprived from their ligand(s), dependence receptors can trigger and/or amplify apoptosis.^24, 26^ Dependence receptors function as caspase substrates leading to proteolytic cleavage, conformational change, exposure/release of an addiction/dependence domain (ADD) and executioner caspases amplification. There are currently more than a dozen identified members of the dependence receptors family known to play critical roles in regulating embryonic development, neurodegeneration and cancer progression.^22, 24, 27^ We previously identified Eph receptors as new members of the dependence receptor family.^21, 22^

Ephrins and Eph receptors are membrane-bound proteins that require cell–cell interactions to activate bi-directional signaling and are known regulators of axonal pathfinding, cell migration and positioning, dendritic spine modeling and angiogenesis during development.^28, 29, 30, 31^ In the adult CNS, EphB3 receptors regulate neural progenitors and cortical neuron survival following traumatic brain injury (TBI),^22, 27^ supporting a dependence receptor role for EphB3. These studies also revealed that EphB3 could have a broader pro-apoptotic role enhancing the survival of multiple cell types in the injured adult CNS.

Here, we examined whether EphB3 mediates OL apoptosis during the acute and chronic phase of contusive SCI through dependence receptor mechanism, and whether administration of ephrinB3 could block EphB3-mediated cell death. Our findings provide further evidence that EphB3 functions as a dependence receptor in the injured CNS and support a potentially important and novel therapeutic strategy to promote recovery.

## Results

### EphB3 is expressed in OLs and is altered after SCI

**Figure 1:**
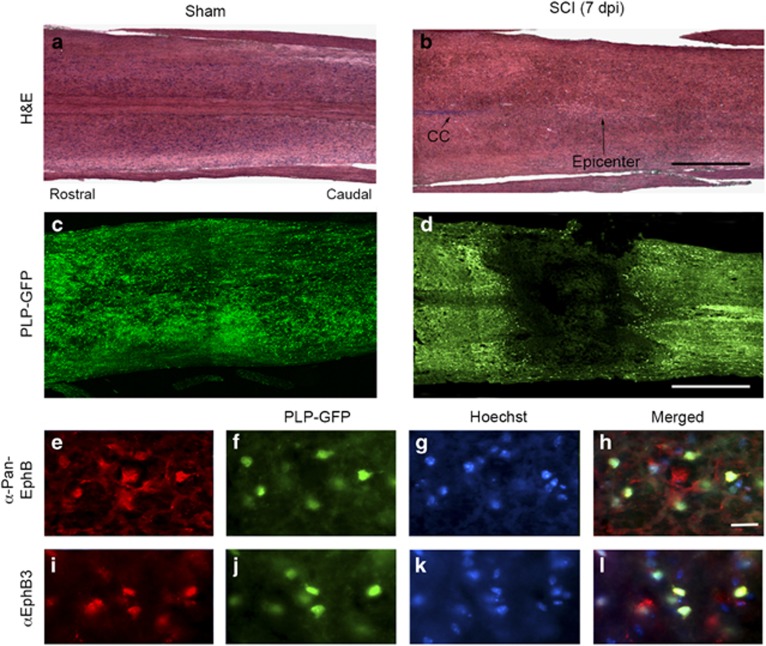
H&E stained longitudinal sections from the spinal cords of sham (**a**) and SCI (**b**) mice showing mild atrophy and morphological tissue disorganization without significant cavitation 1 week after SCI. Conversely, there is an overt loss of PLP-GFP positive OLs at the injury epicenter (**d**) as compared with sham (**c**) mice 7 days after surgery. High-magnification images show double PLP-GFP and EphB positive OLs with Hoechst stained nuclei using a pan-EphB1,2,3 antibodies (**e**–**h**) and specifically EphB3 using anti-EphB3 antibodies (**i**–**l**) in the adult naïve spinal cord. Scale bars equal 250 *μ*m ( × 5 magnification) (**a**–**d**) and 50 *μ*m ( × 63 magnification) (**e**–**l**)

To examine the role of EphB3 in OL survival following SCI, we took advantage of genetically modified mice expressing green fluorescent protein (GFP) under the control of the proteolipid protein (PLP) promoter.^32^ Co-labeling studies demonstrated that GFP was restricted to MBP-labeled OLs and not present in GFAP-positive astrocytes or NeuN-positive neurons (Supplementary Figure S1). PLP-GFP mice were examined at 7 days post injury (dpi) for OL survival and tissue morphology. At this post-surgery time point, haematoxylin and eosin stained sections from SCI animals showed cellular disorganization, but little overt tissue loss at the injury epicenter as compared with sham controls (Figures 1a and b). GFP-positive OLs were abundant throughout the spinal cord under normal physiological conditions (sham controls) (Figure 1c); however, OLs were not observed at the injury site (Figure 1d) suggesting that most OLs in and around the injury epicenter have likely undergone cell death by 7 dpi.

**Figure 2:**
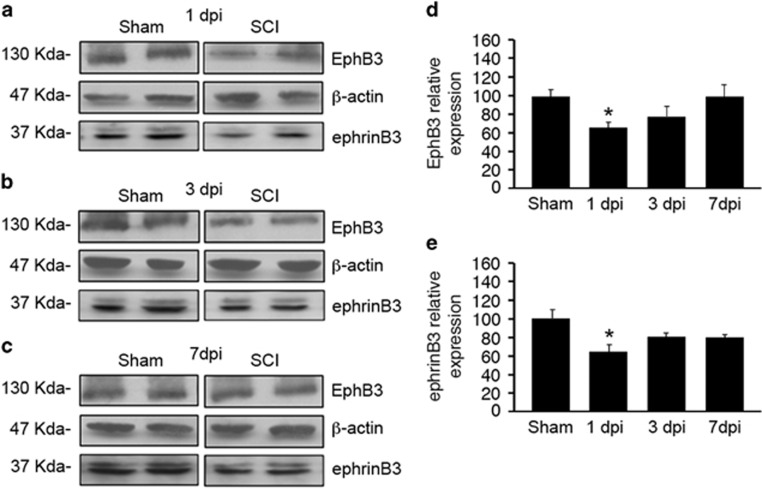
EphB3 and ephrinB3 levels are altered after SCI. Western blot analysis for EphB3 and ephrinB3 at 1 (**a**), 3 (**b**) and 7 (**c**) day(s) post-injury (dpi) as compared with sham surgery. Significant reductions in EphB3 (**d**) and ephrinB3 (**e**) protein levels are observed at 1 dpi with a subsequent trends back to pre-injury levels of the EphB3 receptor, but not the ephrinB3 ligand by 7 dpi. Protein levels are normalized to *β*-actin controls. *P<0.05

To begin implicating Eph receptors in mediating cell death, we first examined whether GFP-positive OLs express Eph receptors in the adult spinal cord. EphB receptors and specifically EphB3 were expressed in GFP-positive OLs using pan-EphB (Figures 1e–h) and anti-EphB3 antibodies (Figures 1i–l), respectively. Furthermore, Eph expression was maintained in both naive and injured tissues. We next determined the temporal protein level profiles of EphB3 and its ligand ephrinB3 in a 2-mm spinal cord segment flanking the injury epicenter at 1, 3 and 7 dpi in WT sham and SCI tissues (Figure 2). We observed a significant reduction in overall EphB3 and ephrinB3 levels at 1 dpi (*P*<0.05) (Figures 2a and d). By 3–7 dpi, EphB3 levels gradually returned to baseline (sham controls) whereas ephrinB3 remained suppressed at the same post-injury time point (Figures 2b, c and e). Together, these findings demonstrate that EphB3 is expressed in spinal cord OLs and may participate in Eph-mediated cell death. This could result from suppression in injury-induced ephrinB3 protein levels, tissue disruption associated with SCI and/or alterations in cell–cell contacts important for the interactions of membrane-bound ephrins with their Eph receptors.

### Improved survival of OLs after SCI in the absence of EphB3 and following ephrinB3 infusion

**Figure 3:**
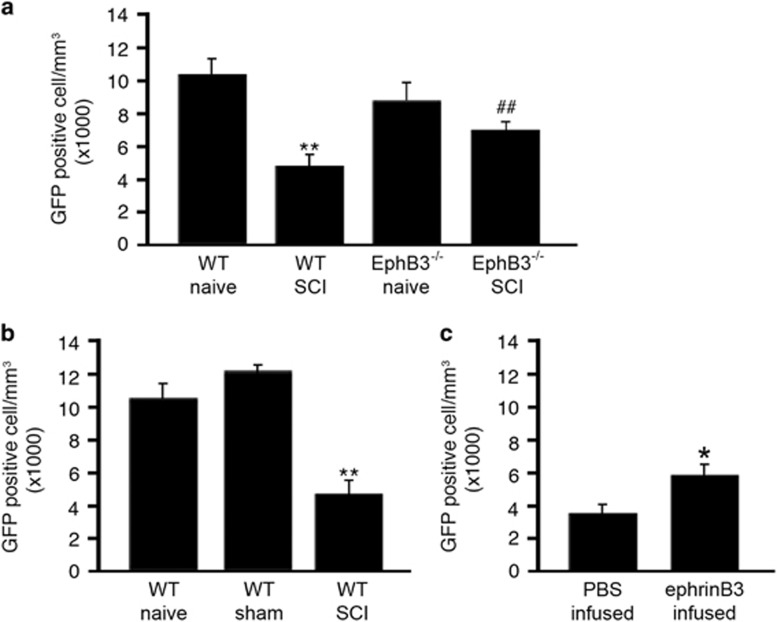
Stereological analysis of PLP-GFP-positive OLs demonstrates that the significant losses observed in WT SCI are not observed in EphB3^−/−^ injured mice as compared with naïve controls with significantly greater EphB3^−/−^ than WT OL numbers at 7dpi (**a**). Sham surgery does not affect overall OL numbers as compared with naïve animals; however, SCI is associated with significant reductions in GFP-positive OLs when compared with sham and naïve controls (**b**). EphrinB3 administration significantly promotes OL survival 1 week after SCI as compared with PBS infusion in SCI tissues (**c**). *P<0.05; **P<0.01; ^##^*P*<0.01. *compared with WT naïve (a) or sham (b); ^#^compared with WT SCI

To determine whether ephrinB3-EphB3 signaling has a role in cell survival following SCI, OL numbers were analyzed by stereologically counting GFP-positive cells at 7 dpi. We first examined OL numbers in naïve PLP-GFP-WT and PLP-GFP-EphB3^−/−^ mice to rule out potential developmental deficits associated with the germ-line mutation, and observed no significant differences in OL numbers between PLP-GFP-WT (10 313±905, *n*=8) and PLP-GFP-EphB3^−/−^ (8851±1312, *n*=9) mice (*P*>0.05) (Figure 3a). This suggests that the absence of EphB3 does not affect OL differentiation, maturation and overall numbers in the adult spinal cord. We next examined the effects of SCI on OL survival within the first week after SCI by evaluating the number of surviving GFP-positive cells in a 2-mm segment surrounding the injury epicenter. At 7 dpi, we observed an ~50% reduction in GFP-positive OLs in PLP-GFP-WT SCI (4903±710, *n*=12) as compared with naïve (10 313±905) and sham (12 138±271, *n*=3) controls (*P*<0.01) (Figures 3a and b). Interestingly, analysis of injured PLP-GFP-EphB3^−/−^ mice (6871±509, *n*=3) showed no significant differences from their non-injured PLP-GFP-EphB3^−/−^ controls (*P*>0.05), but demonstrated significant differences from PLP-GFP-WT SCI mice at 7 dpi (*P*<0.01) (Figure 1a).

To examine whether activation of EphB3 following SCI could reverse cell losses, we administered recombinant ephrinB3^33^ at a concentration of 50 ng/h through intrathecal infusion into the injury epicenter for a week after the initial insult. Following ephrinB3 infusion, we observed a significantly higher number of GFP-positive OLs (5870±742, *n*=6) as compared with vehicle controls (3547±510, *n*=14) (*P*<0.05) (Figure 3c). These findings support the dependence receptor role of EphB3 in mediating OL death following SCI and demonstrate that these negative effects can be reversed by administration of its ligand ephrinB3.

### Absence of EphB3 leads to improved functional recovery following SCI

**Figure 4:**
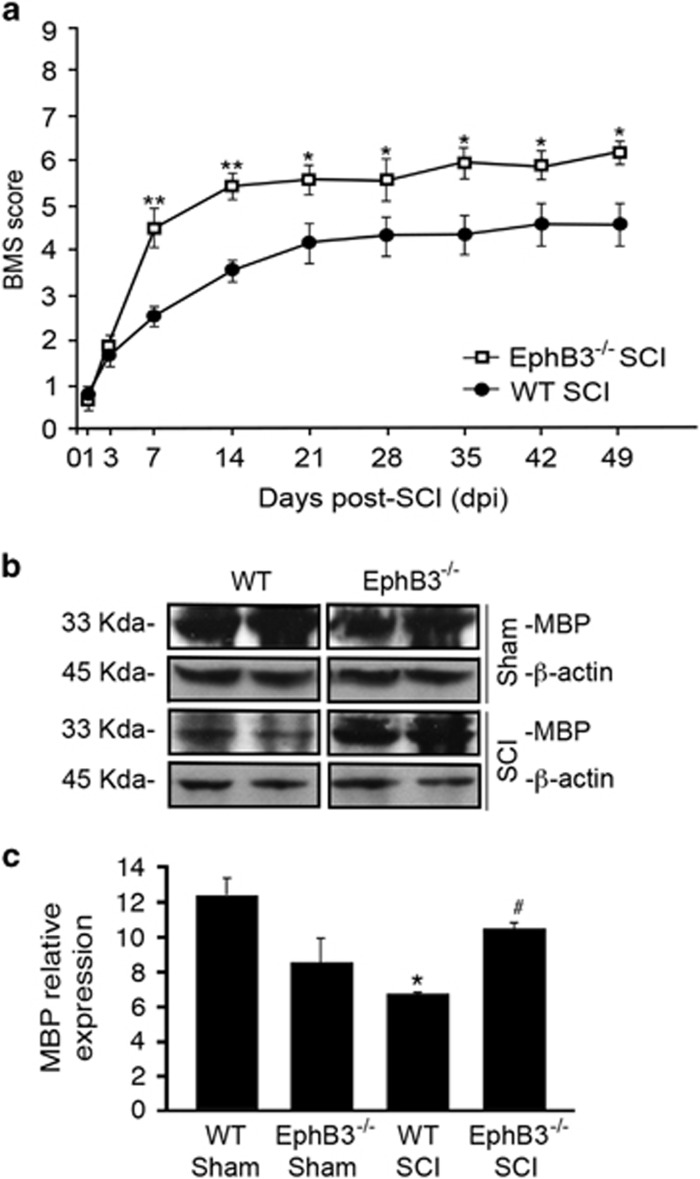
Significant improvements in hindlimb locomotor behavior are observed in EphB3^−/−^ as compared with WT mice by 7 dpi that is maintained for 7 weeks (**a**). Western blot analysis of MBP and *β*-actin in WT and EphB3^−/−^ sham and SCI mice at 7 dpi (**b**). Significant reductions in MBP levels were observed in WT, but not in EphB3^−/−^ SCI tissues, as compared with sham controls when normalized to *β*-actin (**c**). MBP levels in EphB3^−/−^ spinal cord were significantly higher than WT SCI mice. *P<0.05; **P<0.01; ^#^*P*<0.05. *Compared with WT sham; ^#^Compared with WT SCI

We next examined whether increased OL survival resulted in improved hindlimb locomotion and myelination following SCI. To examine hindlimb function, we performed an open field basso mouse scale (BMS) analysis on WT and EphB3^−/−^ mice for a period of 7 weeks after SCI. Both WT and EphB3^−/−^ naïve and sham mice showed normal locomotion before surgery with an average BMS score of 9 (not shown). Following SCI, both genotypes developed severe motor deficits that showed improvements by 3 dpi and plateaued at 14 dpi, which is a standard response for moderate-contusion injury in mice.^34^ Significant differences were observed between WT (average BMS score of ~4) and EphB3^−/−^ (average BMS score of ~5.8) mice between 14 and 49 dpi with the earliest significant improvements occurring by 7 dpi (*P*<0.05) (Figure 4a). It is likely that the motor deficits observed at 1 and 3 dpi represent spinal shock from the surgery, while genotype differences in injury progression occur at 7 dpi and beyond.

To provide additional support for the sparing of mature OLs in the presence and absence of EphB3 following SCI, we examined MBP protein levels at 7 dpi (Figures 4b and c). Western blot analysis showed a significant loss of MBP in WT injured mice at 7 dpi as compared with WT sham controls (*P*<0.05); however, MBP levels in EphB3^−/−^ injured tissues were not significantly different from WT or EphB3^−/−^ sham controls (*P*>0.05). Furthermore, significant differences in MBP levels were observed between WT and EphB3^−/−^ SCI tissues (*P*<0.05) (Figures 4b and c), suggesting that losses of EphB3 expressing OLs may lead to reduced myelin content after SCI.

### EphrinB3 rescues cell death of cultured OPCs and mature OLs

**Figure 5:**
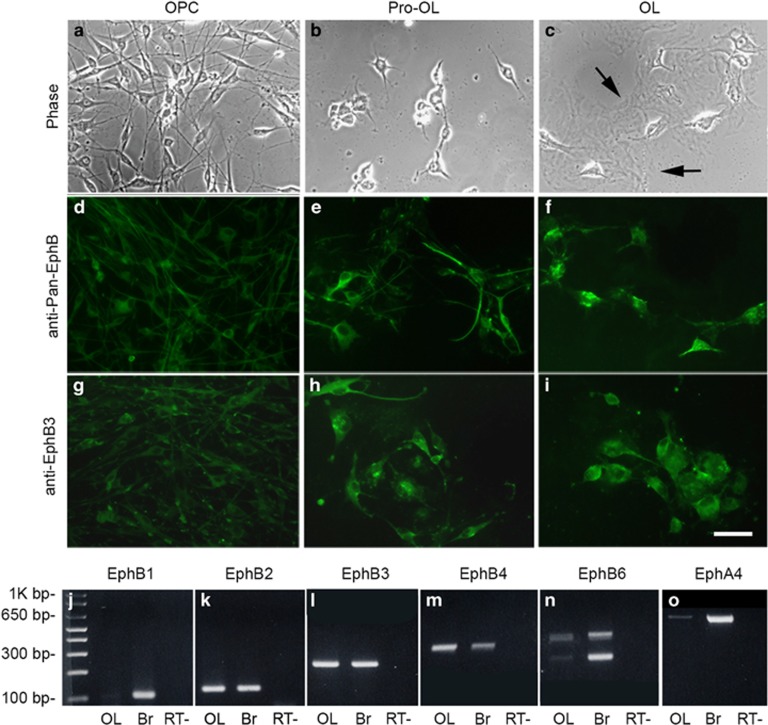
Cultured OLs express Eph receptors in progenitor, pro-OL and mature OL stages. Bipolar progenitors show an elongated soma (**a**), pro-OLs have a larger soma with shorter, more complex branching (**b**), and mature OLs have multiple processes containing membrane sheets (arrows in **c**). High-magnification images show EphB expression at all stages of OLs maturation *in vitro* using anti-pan-EphB1,2,3 (**d**–**f**) and anti-EphB3 antibodies (**g**–**i**). RT-PCR analysis reveals mRNA expression of EphB1 (**j**), EphB2 (**k**), EphB3 (**l**), EphB4 (**m**), EphB6 (**n**) and EphA4 (**o**) in cultured mature OLs derived from the adult spinal cord. mRNA from neonatal brain (Br) was used as a positive control and RT- served as a negative control

To determine whether ephrinB3 can block Eph-mediated cell death in a cell autonomous manner, we used *in vitro* cultured OPCs and mature OLs isolated from the adult spinal cord of naïve WT and EphB3^−/−^ mice. Western blot analysis for EphB3 confirmed the absence of EphB3 protein in cultured OPCs derived from EphB3^−/−^ animals (Supplementary Figure S3D). Cells were maintained as A2B5-positive, O1-/O4-negative progenitor cells before inducing differentiation to O1-/O4-/GalC-positive mature OLs (Supplementary Figure S2). Immunostaining showed that Eph receptors were expressed by all cultured OL subtypes (i.e., progenitor, pro-OLs and mature OLs) using anti-pan-EphB (Figures 5d and f) and anti-EphB3 (Figures 5g–i) antibodies. mRNA analysis demonstrated that multiple Eph transcripts were expressed by mature OLs (Figures 5j–o), including EphB3 (Figure 5l).

**Figure 6:**
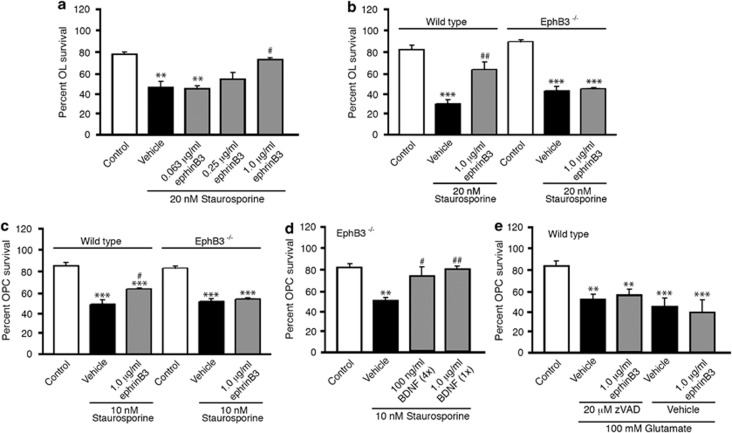
EphrinB3 administration blocks EphB3-mediated cell death in OLs and OPCs. Dose-response curve of 0.063; 0.25; 1.0 *μ*g/ml ephrinB3 blocked 20 nM staurosporine-mediated cell death of WT OLs at 1.0 *μ*g/ml (**a**). Application of 1.0 *μ*g/ml ephrinB3 blocked 20 nM staurosporine-mediated cell death in WT but not EphB3^−/−^ OLs cultures (**b**). Application of 1.0 *μ*g/ml ephrinB3 blocked 10 nM staurosporine-mediated cell death in WT but not EphB3^−/−^ OPCs (**c**). BDNF administration at both 100 ng/ml supplemented every 12 h over 2 days and 1.0 *μ*g/ml applied once over 48 h significantly increased cell survival of staurosporine-treated EphB3^−/−^ OPCs (**d**). 100 mM Glutamate induced significant cell death in WT OPC cultures, which could not be rescued by application of 20 *μ*M zVAD-fmk in combination or not with 1.0 *μ*g/ml ephrinB3 (**e**). **P<0.01; ***P<0.001; ^#^*P*<0.05; ^##^*P*<0.01. *Compared with control; ^#^Compared with vehicle

To begin to determine whether ephrinB3 can block OL cell death in stressed-related conditions, we developed a cell death assay using staurosporine as previously described.^21^ Although the mechanism of staurosporine actions is not well understood, stauroporine has been shown to partially function through caspase-dependent mechanisms,^35, 36^ which is a prerequisite initiation step for inducing dependence receptor cleavage in the absence of ligand binding. Administration of 4–20 nM staurosporine to cultured OLs and OPCs showed a dose-dependent decrease in cell survival (Supplementary Figures S3A and B), where 20 and 10 nM represented the optimal minimal concentration of staurosporine to induce a significant ~50% reduction in OL and OPC survival, respectively. To examine whether application of ephrinB3 could reverse staurosporine-induced cell death, we applied 0.063–1.0 *μ*g/ml ephrinB3 with 20 nM staurosporine to OL cultures (Figure 6a). We observed a dose-dependent increase in OL survival following ephrinB3 administration, where 1.0 *μ*g/ml ephrinB3 significantly blocked staurosporine-induced cell death. To examine whether ephrinB3 regulates OL survival through EphB3, we evaluated the effects of ephrinB3 treatment on WT and EphB3^−/−^ OL derived from adult spinal cords. We observed a significant 2-fold survival in OL cultures treated with 1.0 *μ*g/ml ephrinB3 as compared with vehicle (Figure 6b), where ephrinB3/staurosporine-treated OLs did not differ significantly from controls (*P*>0.05). Conversely, ephrinB3 administration had no effect on staurosporine-treated EphB3^−/−^ OLs (Figure 6b), suggesting that EphB3 signaling is important in ephrinB3-mediated survival.

We also examined the influence of ephrinB3 on OPCs and observed similar effects to OLs, where 1.0 *μ*g/ml ephrinB3 improved WT, but had no effect on EphB3^−/−^ OPC survival when grown in the presence of 10 nM staurosporine (Figure 6c). No significant differences were observed in staurosporine-induced cell death between WT or EphB3^−/−^ OPCs (Supplementary Figure S3C). To exclude potential developmental deficiencies in OPC associated with germ-line *EphB3* deletion, we examined whether EphB3^−/−^ OPCs are responsive to alternative trophic signals after staurosporine treatment. Administration of brain-derived neurotrophic factor (BDNF) using a single bolus of 1.0 *μ*g/ml BDNF or 4 applications of 100 ng/ml every 12 h over 2 days, together with 10 nM staurosporine, showed significant improvement in EphB3^−/−^ OPC survival (Figure 6d). We also examined whether ephrinB3 could improve cell survival in non-caspase, glutamate-induced excitotoxicity model of cell death. Application of 100 mM glutamate induced ~50% cell death in cultured OPCs that was not significantly reversed following 1.0 *μ*g/ml ephrinB3 administration in the presence or absence of 20 *μ*M pan-caspase inhibitor zVAD-fmk. Together, these findings support a cell autonomous role for ephrinB3 in blocking EphB3-mediated cell death in both OLs and OPCs.

## Discussion

Progressive loss of OLs following SCI contributes to prolonged demyelination, axonal degeneration, functional impairment, and represents an important cellular target for new neuroprotective therapies. The present study describes a novel role for EphB3 in the contused spinal cord, where it functions as a pro-apoptotic dependence receptor to induce OL loss. In general, dependence receptors initiate cell death signals under stress conditions and reduced interactions of a ligand with its cognate receptor(s).^21, 22^ In the adult spinal cord, EphB3 is localized to several cell types including OLs throughout the rostro-caudal extent. Following SCI, alterations in the EphB3 to ephrinB3 protein ratio along with tissue disruptions and reduced cell–cell interactions support an environment for EphB3 dependence receptor-mediated cell death to occur. In the absence of EphB3 receptors, fewer OLs undergo cell death leading to improved locomotor behavior in EphB3^−/−^ as compared with WT mice following SCI. Similarly, EphB3-mediated OL cell death can be blocked following administration of ephrinB3 to the injured spinal cord. These results are supported by our *in vitro* studies suggesting a cell autonomous role for EphB3 signaling. In short, these findings demonstrate a novel dependence receptor mechanism for OL cell death following SCI, and implicate a ligand-based therapeutic strategy for blocking progressive spinal cord damage.

The dependence receptors family is best known for its roles in development^28, 29, 30, 31, 37^ and cancer biology.^23, 24^ Many dependence receptors regulate critical processes during neurodevelopment such as axonal chemoattraction/repulsion and outgrowth as well as cell migration. In fact, some of the first functions associated with Eph receptors had been midline axonal chemorepulsion and neural crest cell sorting.^28, 29, 30, 31^ The developing nervous system is also refined by a period of naturally occurring cell death mediated partially by some members of the dependence receptors family such as the netrin-1 binding receptors, Deleted in Colorectal Cancer (DCC) and uncoordinated-5 homolog (UNC5).^37^ In these studies, netrin-1 knockout mice exhibited apoptosis of DCC- and UNC5-expressing cells in the embryonic brainstem. Eph receptors are also highly expressed in the developing CNS, but have not been associated with cell death. In the adult CNS, many important developmentally regulated proteins are still present, although expression levels and pattern may have changed. In particular, membrane-bound ephrins and their Eph receptors are maintained at low homeostatic levels in multiple cell types in adult tissues, but expression patterns and levels can change after CNS injury. Studies have shown that several A- and B-class Ephs are upregulated following SCI in rats, while ephrins are maintained or reduced.^38, 39, 40, 41, 42^ Our findings suggest that overall levels of both ephrins and Eph receptors are reduced in the acute stage following SCI in mice, but only Eph receptors return to pre-injury levels after a week, supporting an environment where non-ligated Eph receptors may persist and mediate cell death through dependence receptor mechanisms. More importantly, since ephrins and Eph receptors are membrane bound, reductions in cell–cell contact after CNS trauma will likely perpetuate a dependence receptor-mediated cell death environment. Basically, tissue damage after trauma leads to cell losses that progressively reduce tissue integrity and destabilize cell–cell contacts. We demonstrate that saturation of spinal cord tissues with ephrinB3 can block OL cell death, likely through inhibition of receptors cleavage by ligand binding. We observed similar roles in TBI, where residential cortical neurons^22^ or neural progenitor cells in the subventricular zone^27^ showed differential EphB3 to ephrinB3 expression associated with EphB3-mediated cell death that could be reversed by ephrinB3 administration. Furthermore, cell death results from cell autonomous EphB3 signaling since we can model cell death and ephrinB3 reversal in purified adult OL and OPC cultures. It is less clear whether blocking OL and/or OPC apoptosis in the injured spinal cord also leads to paracrine influences on each other or other cell types. We did observe a BDNF rescuing effect on EphB3^−/−^ OPCs following staurosporine treatment. BDNF is known to induce trophic signals in developing OPCs and OLs^43^ as well as to promote cell survival under pathological conditions such as SCI.^14, 44, 45^ BDNF-induced OPC survival originating from EphB3^−/−^ adult mouse spinal cord demonstrates a lack of developmental defects associated with the germ-line mutation and continued responsiveness of these cells to trophic signals. Interestingly, BDNF can also bind to p75^NTR^, which is expressed by OLs and has been described as a dependence receptor.^25, 46^ However, unlike TrkA and TrkC, TrkB has not been shown to exert dependence receptor functions.^26, 47, 48, 49^

In addition, endogenous oligodendrogensis and remyelination may also influence our observations, since these events have been observed in the injured spinal cord^50, 51, 52, 53^ and we have previously shown that ephrinB3 can stimulate other neural progenitor cell populations in the adult subventricular zone.^27, 54^ Our *in vitro* findings would support the possibility that ephrinB3-EphB3 signaling may improve the survival and possibly expansion of OPCs under pathological conditions. Thus, we cannot rule out that OPCs may have also contributed to the observed functional improvements associated with EphB3^−/−^ injured mice.

Other membrane receptors have also been implicated in early apoptotic responses following CNS trauma, such as the classic death receptor machinery (e.g., TNFR-alpha, FASR, CD95 and p75^NTR^). Classic death receptors require ligand activation to induce cell death signals that involve terminal activation of caspases, unlike dependence receptors that initiate cell death in the absence of their respective ligands. SCI studies have shown that OL apoptosis along degenerating axons is associated with increased FAS, p75^NTR^ and TNF ligand/receptor expression^12, 18^ while CD95 ligand/receptor are upregulated at the injury epicenter.^19^ Although, both CD95 and TNF have essential roles in inducing programmed cell death, therapeutic neutralization of only CD95 has been shown to significantly decrease apoptosis and promote functional recovery after SCI in mice.^19^ FAS receptor activation is also an important player in apoptosis, and has been linked to neuroinflammatory responses and Wallerian degeneration.^19, 20^ Neutralization of FAS ligand as well as genetic mutation of FAS lead to reduced cell death and enhanced functional recovery after SCI.^19^ Interestingly, soluble FAS receptor administration in the injured spinal cord promotes neuronal and OL survival and improves functional outcome.^20^ Similarly, inhibition of EphA7 upregulation limits the extent of apoptosis and improves recovery after SCI; however, its mechanism(s) of action remain(s) unclear.^38^ In addition, to EphB3, we have also implicated EphA4 as a dependence receptor in the CNS;^21^ while most other A- or B-class receptors have not been evaluated.

Activation of pro-apoptotic signals by ligand-dependent death receptors or ligand-independent dependence receptors have signaling cascades that both involve terminal caspases.^16, 24, 26^ EphB3 is known to require intracellular caspase cleavage at amino acid position 849 to induce cell death, where C-terminal cleavage of an ~20-kDa fragment releases an ADD upstream of this cleavage site.^22^ Both EphB3 and EphA4 have been shown to be caspase targets, where D849N mutation of EphB3 or D773/774N mutation of EphA4 can block Eph-mediated cell death.^21, 22^ Detection of the 20kDa fragment in the injured spinal cord has proven challenging considering highly specific antibodies are currently not available and difficult to develop and Eph receptor cleavage is an early diffuse event where C-terminal fragments can be quickly degraded. Future studies will need to develop an inducible and cell-specific non-cleavable D849N knock-in mouse to examine dependence receptor mechanisms following SCI.

The spatiotemporal profile of cell death following SCI involves numerous cell types and likely reflects multiple mechanisms of initiation. In fact, it is difficult to address one cell type without considering the influence of other cells in the spinal cord. OL has an intimate relationship with the axons they wrap, therefore injury-induced axonal degeneration could lead to decreased axonal trophic support contributing to OL loss.^10, 12^ It is difficult to determine whether axonal degeneration precedes or proceeds OL loss, but it is clear that supporting the survival of OLs may have beneficial effect on neurons. In EphB3^−/−^ mice, we observed significant functional improvement in hindlimb movement following SCI as compared with WT-injured mice, which included significantly higher levels of MBP in the cord. However, assessing the effects of OL sparing on functional improvement are challenging, considering these mechanisms are specific to apoptosis and there remains a necrotic epicenter and significant tract damage. This was most evident in the ephrinB3 infused mice where improved hindlimb locomotion was not observed (not shown), which likely reflects a partial protective effect, compared with the absence of EphB3, and a remaining necrotic core. This would suggest that a therapeutic treatment would need to include both anti-apoptotic and anti-necrotic strategies. Our *in vitro* glutamate-induced excitotoxicity study supports the pro-apoptotic role of ephrinB3 and provides an OPC modeling system to evaluate anti-necrotic compounds. In summary, pro-apoptotic receptors may reflect several families with multiple members, suggesting that a complex multi-treatment may be required. However, significant OL survival can be achieved by eliminating or blocking just one member of the dependence receptor family, suggesting that cell survival in the injured CNS depends on the balance between trophic and anti-trophic signals and therefore, tipping the balance to pro-survival may have important beneficial effects.

## Materials and Methods

### Animals

Female wild type (WT), EphB3 knockout (EphB3^−/−^^55^), PLP-GFP-WT (a kind gift of Dr. Wendy Macklin, University of Colorado, Denver CO, USA^32^) and PLP-GFP-EphB3^−/−^ mice (2–3 months of age, weighing 26±6 g) were used for this study. All mice were maintained on a CD1 background, and genotyped using a standard PCR analysis as previously described.^56^ All animal procedures were approved by the University of Miami Animal Use and Care Committee (IACUC).

### SCI and osmotic pump implantation

Mice were randomly allocated to a naïve, sham or SCI group. Before surgery, sham and SCI mice were anesthetized by intraperitoneal (IP) injection of ketamine (100 mg/kg) and xylazine (10 mg/kg). A laminectomy was performed at vertebrate thoracic level 9 (T9) to expose and moderately injure the dorsal surface of the spinal cord by the tip of a contusion device (Infinite Horizon Impactor from Precision Systems and Instrumentation, LLC) at a predetermined impact force of 50 kDynes. Sham-operated animals underwent an identical surgical procedure in the absence of laminectomy and injury. Naïve control mice were not subjected to any surgical intervention.

For intrathecal infusion, purified ephrinB3 extracellular domain proteins (ephrinB3)^33^ at 100 *μ*g/ml or vehicle 1 × phosphate buffer saline (PBS, pH7.4, Gibco, Langley, OK, USA) was infused in the injury epicenter using a micro-osmotic pump (Alzet, Durect Corp, Cupertino, CA, USA; model 1007D) assembled according to the manufacturer's recommendations and connected to Brain Infusion kits #3 (Alzet). Briefly, pumps were filled with PBS or ephrinB3 aggregates (100 *μ*g/ml) ^33^ and primed overnight in PBS at 37 °C. Following the laminectomy, pre-loaded micro-osmotic pumps were stereotaxically placed with the cannula just over the contusion site: two points of glue were placed on the bottom spacer disc to attach the infusion pumps on the vertebrae anteriorly and posteriorly to the laminectomy window. Three spacer discs were added around the cannulas to prevent them from protruding excessively and the removable tabs were cut. The placement of the infusion cannulas was further secured using a Vicryl absorbable suture thread (Ethicon Inc., Piscataway, NJ, USA): the thread was passed through the lateral muscle and over the catheter tube before being gently tied. The skin was then sutured to cover the pumps and infusion cannulas. After surgery, mice were housed individually and received daily subcutaneous injections of lactated Ringer's solution (Hospira Inc., Lake Forest, IL, USA) to prevent fluid loss and gentamicin (40 mg/kg) (Sigma, St Louis, MO, USA) to avoid urinary tract infections. Manual bladder expression was performed twice a day until animals recovered their ability to spontaneously relieve their bladder. Food and water were provided *ad libitum* throughout the entire experimental period.

### Behavioral evaluation

Locomotor behavior was evaluated using the BMS.^34, 57^ Briefly, BMS is a 9-point scale providing a gross indication of locomotion and its different features. Scores from 0 to 2 suggest no hindlimb ankle movement and paralysis, score of 4 represents occasional plantar stepping and no forelimb–hindlimb coordination, score of 6 is indicative of frequent or consistent plantar stepping and some locomotor coordination, whereas a score of 9 indicates a normal locomotion. Baseline level of performance was measured before surgery in all animals and then locomotion was tested at 1, 3 and 7 dpi followed by a weekly evaluation for a period of 7 weeks.

### Histology

Mice were anesthetized via an IP injection of 0.1 ml ketamine (100 mg/kg) and xylazine (20 mg/kg) cocktail and intracardially perfused with PBS (pH 7.4) followed by fresh cold 4% paraformaldehyde (PFA). One centimeter spinal cord segments flanking the injury epicenter were carefully removed and tissue samples were cryopreserved by an overnight incubation in 30% sucrose solution at 4 °C before embedding in 30% sucrose Tissue-Tek OCT solution (Sakura, Torrance, CA, USA) and isopentane freezing. Thirty micron longitudinal sections were serially cryostat sectioned and stored at −80 °C until used. A standard Hemotoxylin and Eosin (H&E) histological staining was applied to visually examine the overall spinal cord tissue morphology in sham-operated and SCI animals at 7 dpi.

### Immunohistochemistry and microscopy

Tissue sections were rinsed in PBS to remove OCT and post-fixed in fresh 4% PFA for 5 min. Following three PBS washes for 10 min, the samples were permeabilized with 1% Triton X-100 solution in PBS for 30 min at room temperature and blocked in PBS-T (PBS with 0.1% Tween-20) containing 5% heat-inactivated goat serum and 5% bovine serum albumin (BSA) for a period of 2 h. Following serum blocking, tissues were incubated overnight in the respective primary antibody, including anti-GFAP (rabbit polyclonal, Dako, Cambridge, UK, diluted 1 : 750), anti-NeuN (mouse monoclonal, Millipore, Temecula, CA, USA, diluted 1 : 100), anti-pan-EphB (mouse monoclonal that recognizes EphB(1,2,3) receptors, a gift from Dr. Zaven Kaprielian (Albert Einstein College of Medicine, diluted 1 : 5)^58^ and anti-EphB3 (mouse monoclonal, Abcam, Cambridge, UK, diluted 1 : 100) antibodies. The samples were then washed three times for 10 min in PBS-T followed by incubation with the respective Alexa-488- or Alexa-594-conjugated secondary fluorescent antibodies (goat anti-mouse and goat anti-rabbit, Invitrogen, Carlsbad, CA, USA, diluted 1 : 1000 in blocking buffer) for 2 h at room temperature. Following three PBS-T washes for 5 min, cell nuclei were counterstained with Hoescht 33258 (Sigma) at 1.2 *μ*g/ml for 5 min. Subsequently, the samples were washed three times for 5 min with PBS-T and mounted on glass coverslips with Fluoro-Gel (EMSciences, Hatfield, PA, USA). Immunostained sections were visualized using a Zeiss Axiovert 200 M fluorescence microscope (Carl Zeiss, Goettingen, Germany) and images were captured using AxioVision LE V4.5 software (Carl Zeiss) and annotated with Adope Photoshop software.

### Stereology

PLP-GFP-WT (naïve *n*=8; SCI=14) and PLP-GFP-EphB3^−/−^ (naïve *n*=9; SCI *n*=6) mice were killed at 7 dpi. An additional group of PLP-GFP-WT SCI mice was subjected to either 100 *μ*g/ml ephrinB3 (*n*=6) or vehicle PBS (*n*=14) intrathecal administration. All tissue samples were prepared as described above. Stereological counts of PLP-GFP positive cells were performed using a motorized Olympus BX51TRF microscope (Olympus America, Center Valley, PA, USA), Optronix cooled video camera, and MicroBrightField StereoInvestigator software package (MBF Bioscience, Williston, VT, USA). The optical fractionator method and the optical dissector probe were applied to avoid biased cell number estimation. The region of interest spanning around T8–T10 (1 mm rostral and 1 mm caudal to injury epicenter) was manually overlaid with contours using × 10 magnification. A grid of 100 × 100 *μ*m was placed over the selected area and the number of PLP-GFP positive cells was randomly counted using optical fractionator at × 63 magnification (sampling box 50x50 *μ*m).

### Protein assay

EphB3 and ephrinB3 protein levels were measured in 2 mm spinal cord segment flanking the injury epicenter in WT sham (*n*=9) and SCI animals at 1 (*n*=4), 3 (*n*=6) and 7 (*n*=3) dpi. Myelin protein levels namely myelin basic protein (MBP) were assayed using WT and EphB3^−/−^ sham (WT *n*=3; EphB3^−/−^ *n*=3) and SCI (WT *n*=4; EphB3^−/−^ *n*=4) mice at 7 dpi. Spinal cords were rapidly removed and placed in cold 500 *μ*l RIPA buffer (pH 7.5, 1% NP-40, 1% sodium-deoxycholate, 0.1% SDS, 0.15 M NaCl, 2 mM EDTA and 0.01 M sodium phosphate) supplemented with protease (Roche, Florence, SC, USA) and phosphatase (Sigma) inhibitors. Protein lysates were centrifuged at 4 °C at 13 200 r.p.m. for 10 min and the supernatant was collected. Protein concentration was determined by the Lowry assay (Pierce Biotechnology, Rockford, IL, USA) and measured using Life Science UV/VIS DU 530 Spectrophotometer (Beckman Coulter Inc., Miami, FL, USA). Protein samples were resolved on 10% (EphB3 and ephrinB3 assays) and 12% (MBP assay) SDS-PAGE gels and transferred onto nitrocellulose membranes that were subsequently blocked with 5% milk in TBS-T buffer (20 mM Tris, 137 mM NaCl, 0.1% Tween) for 30 min. The primary antibodies anti-EphB3 (mouse monoclonal; Abcam; diluted 1 : 1000), anti-ephrinB3 (goat polyclonal; Santa Cruz Biotechnology Inc., Dallas, TX, USA; diluted 1 : 200), anti-MBP (mouse monoclonal, Abcam, diluted 1 : 100) and anti-*β*-actin (mouse monoclonal; Cell Signaling, Danvers, MA, USA; diluted 1 : 5000) were diluted in 5% milk in TBS-T for overnight incubation at 4 °C. Following this, the membranes were washed three times in TBS-T (5 min/wash) and the corresponding secondary HRP-conjugated antibody (Jackson ImmunoResearch Inc. Laboratories, West Grove, PA, USA, diluted 1 : 5000) was applied for 1 h at room temperature. Blots were developed using SuperSignal West Pico Chemiluminescent Substrate (Pierce Biotechnology) and densitometrically analyzed with One Quantity software (Bio- Rad, Hercules, CA, USA). The level of protein expression was normalized to *β*-actin and then represented as a percentage of sham control levels (for EphB3 and ephrinB3) or as a fold protein change (for MBP).

### Oligodendroglia isolation and *in vitro* cultures

This procedure was modified from an existing protocol.^59^ Spinal cords were dissected from naïve adult WT and EphB3^−/−^ mice, the meninges and dorsal root ganglia removed, and the spinal cord was then rinsed twice in ice-cold DMEM (Gibco). The tissue was chopped with microscissors and digested in 10 ml volume of dissociation buffer (10 U/ml papain (Sigma), 5.5 mM cysteine (Sigma), 2.5 mM EDTA (Sigma), 20 mM HEPES (Sigma), 0.01 N NaOH in Hanks' Balanced Salt Solution (HBSS, Invitrogen)), for 15 min at 37 °C. Dissociated spinal cords were triturated with a 5-ml pipette, then with a 2-ml pipette after another 15 min. The dissociation was stopped by adding an equal volume of stop buffer (250 U/ml DNAse I (Sigma), 0.2% BSA (Sigma), 10 *μ*g/ml gentamicin (Sigma) and 20 mM HEPES (Sigma), pH 7.4 in HBSS)) for 15 min at 37 °C. The dissociated spinal cords were then filtered through a 100-*μ*m filter mesh (BD Biosciences, San Jose, CA, USA) followed by a 40-*μ*m filter mesh (BD Biosciences). The cells were spun at 1500 r.p.m. for 5 min, resuspended in 10% Percoll (GE Healthcare, Pittsburgh, PA, USA) in DMEM, applied on top of a discontinuous 15%/60% Percoll gradient, and spun at 30 000 g for 30 min at 4 °C. The 15%/60% cloudy interface was removed and washed twice with 10 ml of DMEM followed by a spin at 1500 r.p.m. for 5 min at 4 °C. The cells were then resuspended in DMEM with 10% BSA and 10 *μ*g/ml gentamicin (Sigma) and cultured on 100 *μ*g/ml poly-d-lysine (Sigma)-coated 12-well plates. After 2 days of *in vitro* culture, cells were rinsed in 1 × HBSS (Invitrogen) and cultured as progenitors for 5 days on 100 *μ*g/ml poly-d-lysine (Sigma)-coated glass coverslips at a density of 10^6^ cells per well in DMEM supplemented with 1% N2 supplement (Invitrogen), 2% B-27 supplement (Gibco), 1% gentamicin (Sigma), 7.5% NaHCO3 (Sigma), 0.01% BSA (Invitrogen), 10 ng/ml PDGF- AA (Peprotech, Rocky Hill, NJ, USA), 10 ng/ml FGF2 (Peprotech). The expression levels of EphB3 were verified in WT and mutant OPCs by a standard western blot analysis (Supplementary materials; Supplementary Figure S3D). To produce mature OLs, the medium was changed to DMEM supplemented with 1% gentamicin (Sigma), 20 nM thyroid hormone (T3) (Millipore) and 10 ng/ml ciliary neurotrophic factor (CNTF) (Peprotech) for a period of 7–10 days.

For some experiments, OPCs and mature OLs from both genotypes (WT *versus* EphB3^−/−^) were subjected to 48 h treatment with 4, 10 or 20 nM staurosporine (Cell Signaling) to develop and optimize an *in vitro* pro-apoptotic assay (Supplementary materials; Supplementary Figure S3). Subsequently, the optimal staurosporine dose (10 nM for OPC and 20 nM for mature OLs) was applied and the cells were treated with either the vehicle buffer or 0.0625, 0.25, 1 *μ*g/ml ephrinB3 or 100 ng/ml BDNF every 12 h over 2 days. Alternatively, 1.0 *μ*g/ml BDNF was administered as a signal bolus. Glutamate excitoxicity study was performed by applying 100 mM glutamate (Sigma) and evaluating cell death at 48 h as compared with vehicle. The single administration of pan-caspase inhibitor 20 *μ*M ZVAD-fmk (Kamiya Biomedical Company, Seattle, WA, USA) was applied in combination with 1.0 *μ*g/ml ephrinB3 every 12 h for 2 days. Cell death assay was performed by means of a trypan blue (Sigma) exclusion assay and cell counts were performed using an automatic cell counter (Bio-Rad). All *in vitro* experiments were repeated in triplicates. Immunostaining for primary OPC and OL cultures employed anti-A2B5 (Millipore, diluted 1 : 100), -O4, -O1, -GalC antibodies (Gift from Dr. Pat Wood) from hybridomas grown in Iscove's Modified Dulbecco's Media (Gibco) containing 20% fetal clone I serum (Hyclone), 15% hybridoma cloning factor (Fisher), anti-pan-EphB (Gift for Dr. Zaven Kaprielian^58^), and anti-EphB3 (mouse monoclonal, Abcam, diluted 1 : 100) with respective Alexa-488 or Alexa-594 conjugated secondary fluorescent antibodies as described above.

### RT-PCR analysis

RNA was isolated from newborn mouse brains (postnatal day 0) and adult mouse OL cultures (prepared as described above) using the TRIzol reagent (Invitrogen) according to the manufacturer's protocol. The cDNA was generated using the ImPromII RT Kit (Promega, Madison, WI, USA) and the PCR amplifications were performed using the primer pairs presented in Table 1.^60^

### Statistical analysis

Data was analyzed using GraphPad Prism, version 4 (GraphPad Software Inc., San Diego, CA, USA). Unpaired Student's two-tailed *t-*test with Welch's correction was applied for comparisons of two experimental groups. Multiple group comparisons were done using one-way ANOVA followed by Bonferroni *post hoc* test in the case of significance. The BMS behavioral data were analyzed using two-way ANOVA with group (WT SCI *versus* EphB3^−/−^ SCI) × day as factors. Significant group differences were reported for *P*<0.05.

## Figures and Tables

**Table 1:** Primer pairs with their corresponding annealing temperature used for RT-PCR detection of EphB1, EphB2, EphB3, EphB4, EphB6, EphA4 mRNA levels

**Gene**	**Primers**	**Annealing temperature (**°C**)**	**Product length (bp)**
EphB1	Sense: atccggaacccagctagtctcaag	55	102
	Antisense: ggtggtaaaggccgtgaagtctg		
EphB2	Sense: accaccggcccaagttcg	55	142
	Antisense: aagctggtgtagtccggtatcg		
EphB3	Sense: gtttatattgacccttttaccta	55	242
	Antisense: gtcgaccacggcacacttcc		
EphB4	Sense: gaatcccgctagcctcaaaa	57	328
	Antisense: tcagaactgctgggctggtc		
EphB6	Sense: gttccggctgcccccacctc	55	279
	Antisense: gagaagttgtcctcctggtagcac		
EphA4	Sense: aggaagtgagcattatggatga	55	643
	Antisense: tgctcctcgtgcccagcgtt		

## Abbreviations

ADD: addiction/dependence domain
BDNF: brian-derived neurotrophic factor
BMS: Basso mouse scale
BSA: bovine serum albumin
CNS: central nervous system
CNTF: ciliary neurotrophic factor
Dpi: days post-injury
DCC: deleted in colorectal cancer
DMEM: Dulbecco's Modified Eagle Medium
EDTA: ethylenediaminetetraacetic acid
FGF2: basic fibroblast growth factor 2
GalC: galactosylceramidase C
GFAP: glial fibrillary acidic protein
GFP: green fluorescent protein
HBSS: Hank's balanced salt solution
H&E: hemotoxylin and eosin
HEPES: 4-(2-hydroxyethyl)-1-piperazineethanesulfonic acid
IP: intraperitoneal
MBP: myelin basic protein
OL: oligodendrocyte
OPC: oliogodendrocyte progenitor cell
PBS: phosphate-buffered saline
PBS-T: phosphate-buffered saline with Tween20
PDGF- AA: platelet-derived growth factor A
PFA: paraformaldehyde
PLP: proteolipid protein
SCI: spinal cord injury
T3: thyroid hormone
T9: thoracic level 9
TBI: traumatic brain injury
TNF: tumor necrosis factor
TNFR1: tumor necrosis factor receptor 1
Unc5: uncoordinated homolog 5
WT: wild type

## References

[bib1] 1Lee BB, Cripps RA, Fitzharris M, Wing PC. The global map for traumatic spinal cord injury epidemiology: update 2011, global incidence rate. Spinal Cord 2014; 52: 110–116. 23439068 10.1038/sc.2012.158

[bib2] 2Liu XZ, Xu XM, Hu R, Du C, Zhang SX, McDonald JW et al. Neuronal and glial apoptosis after traumatic spinal cord injury. J Neurosci 1997; 17: 5395–5406. 9204923 10.1523/JNEUROSCI.17-14-05395.1997 PMC6793816

[bib3] 3Wada S, Yone K, Ishidou Y, Nagamine T, Nakahara S, Niiyama T et al. Apoptosis following spinal cord injury in rats and preventative effect of N-methyl-D-aspartate receptor antagonist. J Neurosurg 1999; 91: 98–104. 10419375 10.3171/spi.1999.91.1.0098

[bib4] 4Beattie MS. Inflammation and apoptosis: linked therapeutic targets in spinal cord injury. Trends Mol Med 2004; 10: 580–583. 15567326 10.1016/j.molmed.2004.10.006

[bib5] 5Teng YD, Choi H, Onario RC, Zhu S, Desilets FC, Lan S et al. Minocycline inhibits contusion-triggered mitochondrial cytochrome c release and mitigates functional deficits after spinal cord injury. Proc Natl Acad Sci USA 2004; 101: 3071–3076. 14981254 10.1073/pnas.0306239101 PMC365746

[bib6] 6Totoiu MO, Keirstead HS. Spinal cord injury is accompanied by chronic progressive demyelination. J Comp Neurol 2005; 486: 373–383. 15846782 10.1002/cne.20517

[bib7] 7Katoh K, Ikata T, Katoh S, Hamada Y, Nakauchi K, Sano T et al. Induction and its spread of apoptosis in rat spinal cord after mechanical trauma. Neurosci Lett 1996; 216: 9–12. 8892379 10.1016/0304-3940(96)12999-2

[bib8] 8Li GL, Brodin G, Farooque M, Funa K, Holtz A, Wang WL et al. Apoptosis and expression of Bcl-2 after compression trauma to rat spinal cord. J Neuropathol Exp Neurol 1996; 55: 280–289. 8786386 10.1097/00005072-199603000-00003

[bib9] 9Crowe MJ, Bresnahan JC, Shuman SL, Masters JN, Beattie MS. Apoptosis and delayed degeneration after spinal cord injury in rats and monkeys. Nat Med 1997; 3: 73–76. 8986744 10.1038/nm0197-73

[bib10] 10Abe Y, Yamamoto T, Sugiyama Y, Watanabe T, Saito N, Kayama H et al. Apoptotic cells associated with Wallerian degeneration after experimental spinal cord injury: a possible mechanism of oligodendroglial death. J Neurotrauma 1999; 16: 945–952. 10547103 10.1089/neu.1999.16.945

[bib11] 11Li GL, Farooque M, Holtz A, Olsson Y. Apoptosis of oligodendrocytes occurs for long distances away from the primary injury after compression trauma to rat spinal cord. Acta Neuropathol 1999; 98: 473–480. 10541870 10.1007/s004010051112

[bib12] 12Casha S, Yu WR, Fehlings MG. Oligodendroglial apoptosis occurs along degenerating axons and is associated with FAS and p75 expression following spinal cord injury in the rat. Neuroscience 2001; 103: 203–218. 11311801 10.1016/s0306-4522(00)00538-8

[bib13] 13Emery E, Aldana P, Bunge MB, Puckett W, Srinivasan A, Keane RW et al. Apoptosis after traumatic human spinal cord injury. J Neurosurg 1998; 89: 911–920. 9833815 10.3171/jns.1998.89.6.0911

[bib14] 14Koda M, Murakami M, Ino H, Yoshinaga K, Ikeda O, Hashimoto M et al. Brain-derived neurotrophic factor suppresses delayed apoptosis of oligodendrocytes after spinal cord injury in rats. J Neurotrauma 2002; 19: 777–785. 12165137 10.1089/08977150260139147

[bib15] 15Dubreuil CI, Winton MJ, McKerracher L. Rho activation patterns after spinal cord injury and the role of activated Rho in apoptosis in the central nervous system. J Cell Biol 2003; 162: 233–243. 12860969 10.1083/jcb.200301080 PMC2172802

[bib16] 16Ashkenazi A, Dixit VM. Death receptors: signaling and modulation. Science 1998; 281: 1305–1308. 9721089 10.1126/science.281.5381.1305

[bib17] 17Bethea JR, Nagashima H, Acosta MC, Briceno C, Gomez F, Marcillo AE et al. Systemically administered interleukin-10 reduces tumor necrosis factor-alpha production and significantly improves functional recovery following traumatic spinal cord injury in rats. J Neurotrauma 1999; 16: 851–863. 10547095 10.1089/neu.1999.16.851

[bib18] 18Beattie MS, Harrington AW, Lee R, Kim JY, Boyce SL, Longo FM et al. ProNGF induces p75-mediated death of oligodendrocytes following spinal cord injury. Neuron 2002; 36: 375–386. 12408842 10.1016/s0896-6273(02)01005-x PMC2681189

[bib19] 19Demjen D, Klussmann S, Kleber S, Zuliani C, Stieltjes B, Metzger C et al. Neutralization of CD95 ligand promotes regeneration and functional recovery after spinal cord injury. Nat Med 2004; 10: 389–395. 15004554 10.1038/nm1007

[bib20] 20Ackery A, Robins S, Fehlings MG. Inhibition of Fas-mediated apoptosis through administration of soluble Fas receptor improves functional outcome and reduces posttraumatic axonal degeneration after acute spinal cord injury. J Neurotrauma 2006; 23: 604–616. 16689665 10.1089/neu.2006.23.604

[bib21] 21Furne C, Ricard J, Cabrera JR, Pays L, Bethea JR, Mehlen P et al. EphrinB3 is an anti-apoptotic ligand that inhibits the dependence receptor functions of EphA4 receptors during adult neurogenesis. Biochim Biophys Acta 2009; 1793: 231–238. 18948148 10.1016/j.bbamcr.2008.09.009 PMC2631096

[bib22] 22Theus MH, Ricard J, Glass SJ, Travieso LG, Liebl DJ. EphrinB3 blocks EphB3 dependence receptor functions to prevent cell death following traumatic brain injury. Cell Death Dis 2014; 5: e1207. 24810043 10.1038/cddis.2014.165 PMC4047907

[bib23] 23Mehlen P, Rabizadeh S, Snipas SJ, Assa-Munt N, Salvesen GS, Bredesen DE. The DCC gene product induces apoptosis by a mechanism requiring receptor proteolysis. Nature 1998; 395: 801–804. 9796814 10.1038/27441

[bib24] 24Mehlen P, Bredesen DE. The dependence receptor hypothesis. Apoptosis 2004; 9: 37–49. 14739597 10.1023/B:APPT.0000012120.66221.b2

[bib25] 25Goldschneider D, Mehlen P. Dependence receptors: a new paradigm in cell signaling and cancer therapy. Oncogene 2010; 29: 1865–1882. 20173780 10.1038/onc.2010.13

[bib26] 26Tauszig-Delamasure S, Yu LY, Cabrera JR, Bouzas-Rodriguez J, Mermet-Bouvier C, Guix C et al. The TrkC receptor induces apoptosis when the dependence receptor notion meets the neurotrophin paradigm. Proc Natl Acad Sci USA 2007; 104: 13361–13366. 17686986 10.1073/pnas.0701243104 PMC1948910

[bib27] 27Theus MH, Ricard J, Bethea JR, Liebl DJ. EphB3 limits the expansion of neural progenitor cells in the subventricular zone by regulating p53 during homeostasis and following traumatic brain injury. Stem Cells 2010; 28: 1231–1242. 20496368 10.1002/stem.449 PMC2967180

[bib28] 28Pasquale EB. Eph receptor signalling casts a wide net on cell behaviour. Nat Rev Mol Cell Biol 2005; 6: 462–475. 15928710 10.1038/nrm1662

[bib29] 29Goldshmit Y, McLenachan S, Turnley A. Roles of Eph receptors and ephrins in the normal and damaged adult CNS. Brain Res Rev 2006; 52: 327–345. 16774788 10.1016/j.brainresrev.2006.04.006

[bib30] 30Mendes SW, Henkemeyer M, Liebl DJ. Multiple Eph receptors and B-class ephrins regulate midline crossing of corpus callosum fibers in the developing mouse forebrain. J Neurosci 2006; 26: 882–892. 16421308 10.1523/JNEUROSCI.3162-05.2006 PMC6675355

[bib31] 31Rodenas-Ruano A, Perez-Pinzon MA, Green EJ, Henkemeyer M, Liebl DJ. Distinct roles for ephrinB3 in the formation and function of hippocampal synapses. Dev Biol 2006; 292: 34–45. 16466709 10.1016/j.ydbio.2006.01.004

[bib32] 32Fuss B, Mallon B, Phan T, Ohlemeyer C, Kirchhoff F, Nishiyama A et al. Purification and analysis of in vivo-differentiated oligodendrocytes expressing the green fluorescent protein. Dev Biol 2000; 218: 259–274. 10656768 10.1006/dbio.1999.9574

[bib33] 33Nelersa CM, Barreras H, Runko E, Ricard J, Shi Y, Glass SJ et al. High-content analysis of proapoptotic EphA4 dependence receptor functions using small-molecule libraries. J Biomol Screen 2012; 17: 785–795. 22492230 10.1177/1087057112440880 PMC4380140

[bib34] 34Basso DM, Fisher LC, Anderson AJ, Jakeman LB, McTigue DM, Popovich PG. Basso Mouse Scale for locomotion detects differences in recovery after spinal cord injury in five common mouse strains. J Neurotrauma 2006; 23: 635–659. 16689667 10.1089/neu.2006.23.635

[bib35] 35Belmokhtar CA, Hillion J, Segal-Bendirdjian E. Staurosporine induces apoptosis through both caspase-dependent and caspase-independent mechanisms. Oncogene 2001; 20: 3354–3362. 11423986 10.1038/sj.onc.1204436

[bib36] 36Zhang XD, Gillespie SK, Hersey P. Staurosporine induces apoptosis of melanoma by both caspase-dependent and -independent apoptotic pathways. Mol Cancer Ther 2004; 3: 187–197. 14985459

[bib37] 37Llambi F, Causeret F, Bloch-Gallego E, Mehlen P. Netrin-1 acts as a survival factor via its receptors UNC5H and DCC. EMBO J 2001; 20: 2715–2722. 11387206 10.1093/emboj/20.11.2715 PMC125255

[bib38] 38Figueroa JD, Benton RL, Velazquez I, Torrado AI, Ortiz CM, Hernandez CM et al. Inhibition of EphA7 up-regulation after spinal cord injury reduces apoptosis and promotes locomotor recovery. J Neurosci Res 2006; 84: 1438–1451. 16983667 10.1002/jnr.21048

[bib39] 39Irizarry-Ramirez M, Willson CA, Cruz-Orengo L, Figueroa J, Velazquez I, Jones H et al. Upregulation of EphA3 receptor after spinal cord injury. J Neurotrauma 2005; 22: 929–935. 16083359 10.1089/neu.2005.22.929

[bib40] 40Miranda JD, White LA, Marcillo AE, Willson CA, Jagid J, Whittemore SR. Induction of Eph B3 after spinal cord injury. Exp Neurol 1999; 156: 218–222. 10192794 10.1006/exnr.1998.7012

[bib41] 41Willson CA, Irizarry-Ramirez M, Gaskins HE, Cruz-Orengo L, Figueroa JD, Whittemore SR et al. Upregulation of EphA receptor expression in the injured adult rat spinal cord. Cell Transplant 2002; 11: 229–239. 12075988

[bib42] 42Willson CA, Miranda JD, Foster RD, Onifer SM, Whittemore SR. Transection of the adult rat spinal cord upregulates EphB3 receptor and ligand expression. Cell Transplant 2003; 12: 279–290. 12797382 10.3727/000000003108746830

[bib43] 43Du YZ, Fischer TZ, Lee LN, Lercher LD, Dreyfus CF. Regionally specific effects of BDNF on oligodendrocytes. Dev Neurosci 2003; 25: 116–126. 12966210 10.1159/000072261

[bib44] 44Dougherty KD, Dreyfus CF, Black IB. Brain-derived neurotrophic factor in astrocytes, oligodendrocytes, and microglia/macrophages after spinal cord injury. Neurobiol Dis 2000; 7: 574–585. 11114257 10.1006/nbdi.2000.0318

[bib45] 45Xiao JH, Wong AW, Willingham MM, van den Buuse M, Kilpatrick TJ, Murray SS. Brain-derived neurotrophic factor promotes central nervous system myelination via a direct effect upon oligodendrocytes. Neurosignals 2010; 18: 186–202. 21242670 10.1159/000323170

[bib46] 46Rabizadeh S, Ye X, Sperandio S, Wang JJL, Ellerby HM, Ellerby LM et al. Neurotrophin dependence domain - A domain required for the mediation of apoptosis by the p75 neurotrophin receptor. J Mol Neurosci 2000; 15: 215–229. 11303785 10.1385/JMN:15:3:215

[bib47] 47Nikoletopoulou V, Lickert H, Frade JM, Rencurel C, Giallonardo P, Zhang LX et al. Neurotrophin receptors TrkA and TrkC cause neuronal death whereas TrkB does not. Nature 2010; 467: 59–U87. 20811452 10.1038/nature09336

[bib48] 48Genevois AL, Ichim G, Coissieux MM, Lambert MP, Lavial F, Goldschneider D et al. Dependence receptor TrkC is a putative colon cancer tumor suppressor. P Natl Acad Sci USA 2013; 110: 3017–3022. 10.1073/pnas.1212333110 PMC3581924 23341610

[bib49] 49Ichim G, Tauszig-Delamasure S, Mehlen P. Neurotrophins and cell death. Exp Cell Res 2012; 318: 1221–1228. 22465479 10.1016/j.yexcr.2012.03.006

[bib50] 50Rabchevsky AG, Sullivan PG, Scheff SW. Temporal-spatial dynamics in oligodendrocyte and glial progenitor cell numbers throughout ventrolateral white matter following contusion spinal cord injury. Glia 2007; 55: 831–843. 17390308 10.1002/glia.20508

[bib51] 51Barnabe-Heider F, Goritz C, Sabelstrom H, Takebayashi H, Pfrieger FW, Meletis K et al. Origin of new glial cells in intact and injured adult spinal cord. Cell Stem Cell 2010; 7: 470–482. 20887953 10.1016/j.stem.2010.07.014

[bib52] 52Zawadzka M, Rivers LE, Fancy SP, Zhao C, Tripathi R, Jamen F et al. CNS-resident glial progenitor/stem cells produce Schwann cells as well as oligodendrocytes during repair of CNS demyelination. Cell Stem Cell 2010; 6: 578–590. 20569695 10.1016/j.stem.2010.04.002 PMC3856868

[bib53] 53Hesp ZC, Goldstein EA, Miranda CJ, Kaspar BK, McTigue DM. Chronic oligodendrogenesis and remyelination after spinal cord injury in mice and rats. J Neurosci 2015; 35: 1274–1290. 25609641 10.1523/JNEUROSCI.2568-14.2015 PMC4300327

[bib54] 54Ricard J, Salinas J, Garcia L, Liebl DJ. EphrinB3 regulates cell proliferation and survival in adult neurogenesis. Mol Cell Neurosci 2006; 31: 713–722. 16483793 10.1016/j.mcn.2006.01.002

[bib55] 55Orioli D, Henkemeyer M, Lemke G, Klein R, Pawson T. Sek4 and Nuk receptors cooperate in guidance of commissural axons and in palate formation. EMBO J 1996; 15: 6035–6049. 8947026 PMC452425

[bib56] 56Henkemeyer M, Orioli D, Henderson JT, Saxton TM, Roder J, Pawson T et al. Nuk controls pathfinding of commissural axons in the mammalian central nervous system. Cell 1996; 86: 35–46. 8689685 10.1016/s0092-8674(00)80075-6

[bib57] 57Basso DM, Beattie MS, Bresnahan JC. A sensitive and reliable locomotor rating scale for open field testing in rats. J Neurotrauma 1995; 12: 1–21. 7783230 10.1089/neu.1995.12.1

[bib58] 58Jevince AR, Kadison SR, Pittman AJ, Chien CB, Kaprielian Z. Distribution of EphB receptors and ephrin-B1 in the developing vertebrate spinal cord. J Comp Neurol 2006; 497: 734–750. 16786562 10.1002/cne.21001 PMC2637817

[bib59] 59Cohen RI, Rottkamp DM, Maric D, Barker JL, Hudson LD. A role for semaphorins and neuropilins in oligodendrocyte guidance. J Neurochem 2003; 85: 1262–1278. 12753085 10.1046/j.1471-4159.2003.01722.x

[bib60] 60del Valle K, Theus MH, Bethea JR, Liebl DJ, Ricard J. Neural progenitors proliferation is inhibited by EphB3 in the developing subventricular zone. Int J Dev Neurosci 2011; 29: 9–14. 20969945 10.1016/j.ijdevneu.2010.10.005 PMC3004986

