# Supplementary figures and images for: EphB3 receptors function as dependence receptors to mediate oligodendrocyte cell death following contusive spinal cord injury
Source: Cell Death Dis. 2015 Oct 15;6(10):e1922–. doi: 10.1038/cddis.2015.262 (PMC4632292; doi:10.1038/cddis.2015.262)

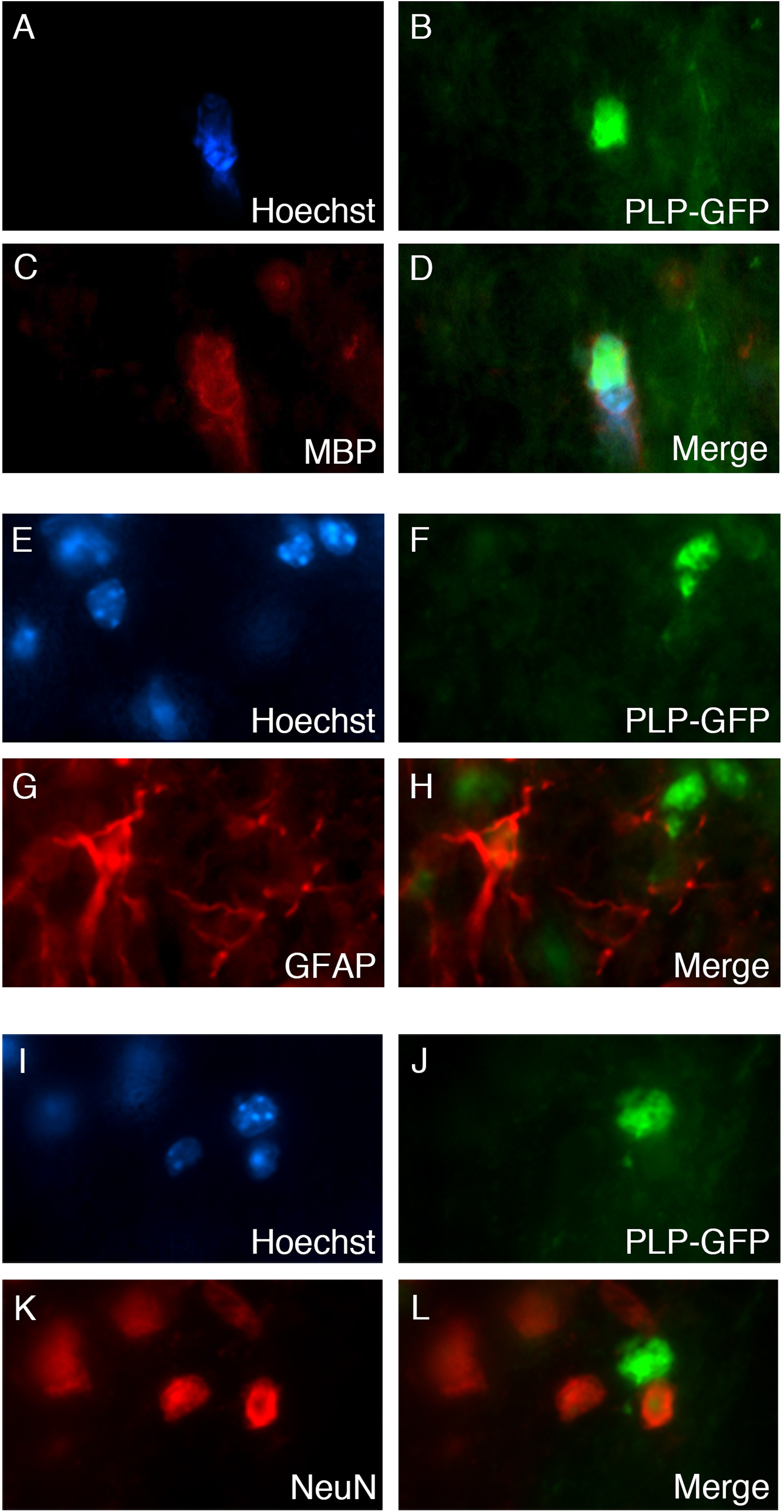

Supplement: Supplementary Figure 1 [file cddis2015262x1.tif]

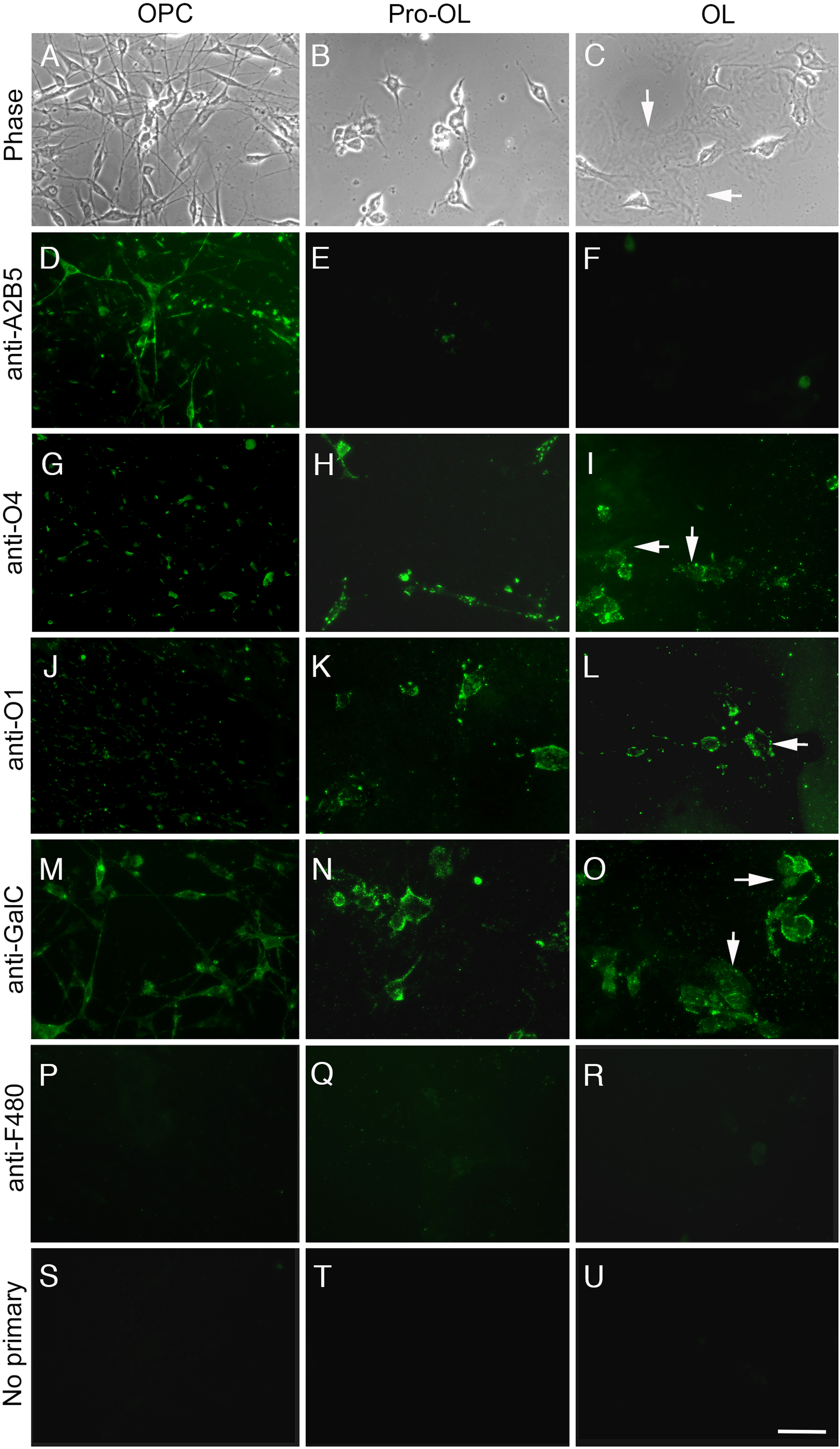

Supplement: Supplementary Figure 2 [file cddis2015262x2.tif]

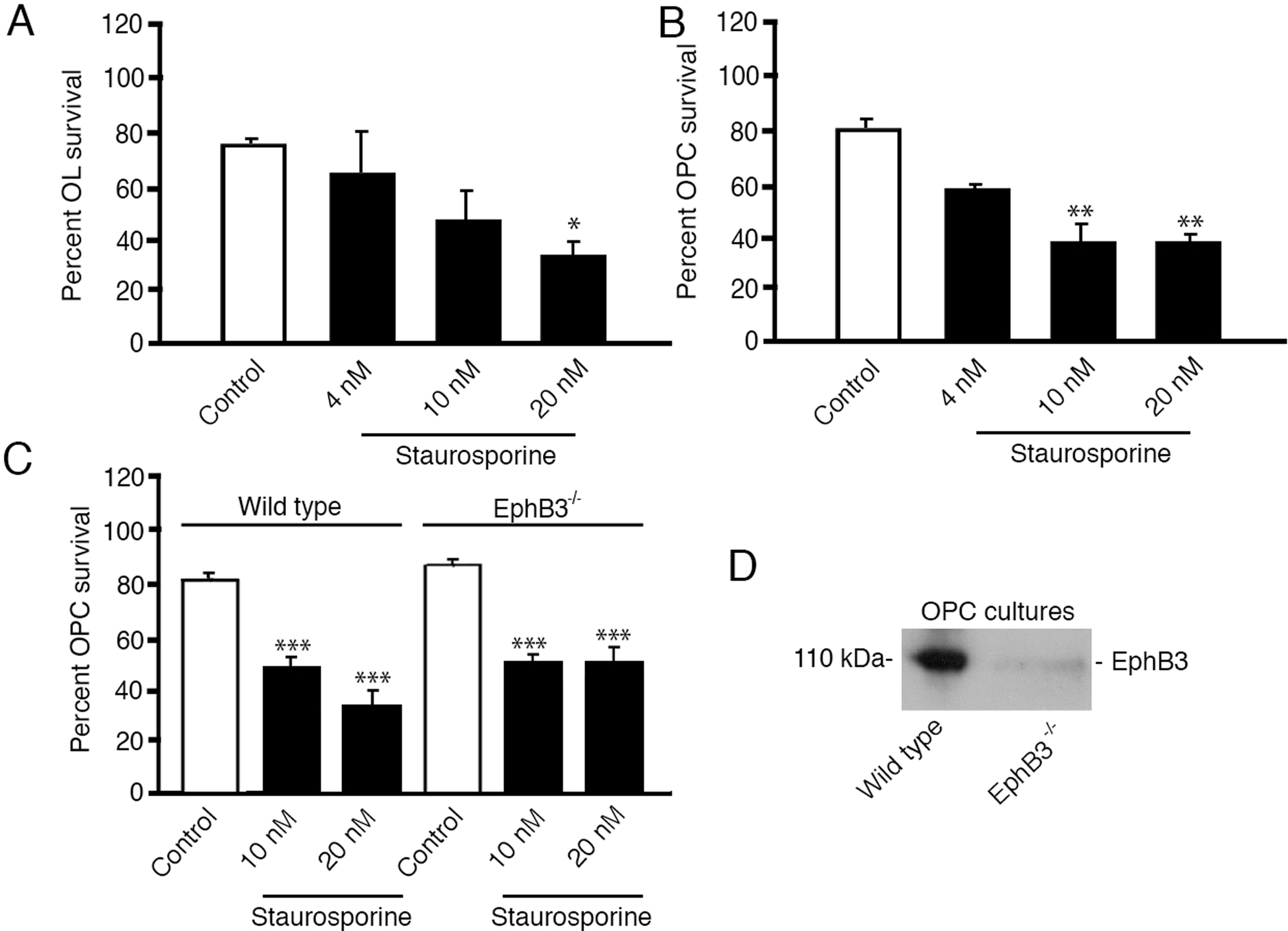

Supplement: Supplementary Figure 3 [file cddis2015262x3.tif]
