# Supplementary material for: EphB3 receptors function as dependence receptors to mediate oligodendrocyte cell death following contusive spinal cord injury
Source: Cell Death Dis. 2015 Oct 15;6(10):e1922–. doi: 10.1038/cddis.2015.262 (PMC4632292; doi:10.1038/cddis.2015.262)
Supplement: Supplementary Figure Legends [file cddis2015262x4.doc]

**Supplementary Figure Legends**

**Figure S1.** High-magnification images of spinal cords from PLP-GFP mice demonstrate colocalization of GFP with MBP (**A**-**D**), but not with GFAP (**E**-**H**) or NeuN (**I**-**L**) labeled cells in the adult naïve spinal cord. Cellular nuclei are counterstained with Hoechst (**A**, **E**, **I**). Scale bar equals 50µm (63X magnification).

**Figure S2.** High-magnification images show specific markers for three stages of cultured OL development, namely OPCs (**A**), pro-OLs (**B**), and mature OLs (**C**). All three stages have characteristic morphologies that include, bipolar progenitors with elongated soma, pro-OLs with larger soma surrounded by more complex branching, and mature OLs with multiple processes containing membrane sheets (arrows in **C**). Progenitors express early markers that include A2B5 (**D**) and GalC (**M**) but not O4 (**G**), O1 (**J**) or the microglial marker F480 (**P**). Pro-OLs and mature OLs express late stage markers that include O4 (**H**), O1 (**K**) and GalC (**N**) but not A2B5 (**E**) or the microglial marker F480 (**Q**). Mature OLs show distinct membrane sheets not observed in pro-OLs (**C**). No primary controls do not show reactivity (**S**-**U**). Scale bar equals 50µm (63 X magnification).

**Figure S3.** Dose-dependence curve of 4 nM, 10 nM, and 20 nM staurosporine treatment in cultured mature OLs (**A**) and OPCs (**B**) for 48 hours show significant cell death at minimal concentrations of 20 nM and 10 nM staurosporine, respectively. WT and EphB3^-/-^ OPCs were treated with 10nM and 20nM staurosporine over 48 hours with significant and similar reductions in cell survival at 48 hours (**C**). Western blot analysis reveals EphB3 protein expression in cultured WT but not EphB3^-/-^ OPCs (**D**). *p<0.05; **p<0.01; ***p<0.001.
